# An intense, few-cycle source in the long-wave infrared

**DOI:** 10.1038/s41598-019-42433-1

**Published:** 2019-04-12

**Authors:** Derrek J. Wilson, Adam M. Summers, Stefan Zigo, Brandin Davis, Seyyed-Javad Robatjazi, Jeffery A. Powell, Daniel Rolles, Artem Rudenko, Carlos A. Trallero-Herrero

**Affiliations:** 1 J. R. Macdonald Laboratory, Department of Physics, Kansas State University, 116 Cardwell Hall, Manhattan, KS 66506 USA; 2 Advanced Laser Light Source and few-cycle Inc., Institut Nationale de la Recherche Scientifique, 1650 Boul. Lionel-Boulet, Varennes, QC J3X 1P7 Canada; 3 Department of Physics, University of Connecticut, 2152 Hillside Road, Unit 3046, Storrs, CT 06269 USA

## Abstract

For the last several decades, the wavelength range accessible for strong-field, few-cycle studies has remained limited to the visible, near infrared and mid-wave infrared regimes. In particular, sources in the long-wave infrared have been lacking. We report the development of a 1 kHz, few-cycle laser source with up to a 9 *μ*m central wavelength and gigawatt peak powers. When focused, this source can ionize gas targets, which we demonstrate here through the ionization of atomic xenon at wavelengths ranging from 5 *μ*m to 9 *μ*m. This opens up new opportunities for fundamental atomic and molecular physics, enabling experimental tests of strong-field ionization theories in the extreme long-wavelength, few-cycle limit and the direct excitation of vibrational transitions in organic molecules.

## Introduction

The interaction of strong laser fields with atoms and molecules has been of considerable interest for decades. Technical developments in this area have led to, among other breakthroughs, the generation of attosecond pulses^1,2^ and provided tools for the imaging of molecular dynamics with sub-femtoscecond temporal^3^ and angstrom spatial resolution^4,5^. These applications would strongly benefit from longer wavelength driving sources as the pondermotive energy of an electron accelerated by a laser field scales with the square of the wavelength. The extension of strong-field, ultrafast science into the MWIR regime^6–9^ has allowed for scientific advances such as the first demonstration of a table-top coherent X-ray source^10,11^, the generation of isolated attosecond pulses with photon energies up to 300 eV^2^, as well as the first femtosecond-resolved measurement of chemical bond dynamics with angstrom resolution^5^.

Such studies have motivated the development of intense ultrafast sources at even longer wavelengths in the long-wave infrared (LWIR, 8–15 *μ*m) regime. The quadratic wavelength scaling of the energy of the photoelectrons presents an enticing reason for pushing to longer wavelengths. Additionally, broadband pulses in this regime allow for the simultaneous excitation of many molecular ro-vibrational energy levels^12,13^. Unlike LWIR sources emphasizing high average power, intense few-cycle fields in the LWIR promise to deliver new strong-field studies in molecular phenomena^14^. Other interesting possibilities with intense, ultrafast sources in this wavelength regime include the observation of dynamics due to the breakdown of the dipole approximation at modest intensities^15,16^ and extreme modification of optical waveforms via shock formation^17^. However, the development of high energy, ultrafast laser sources in the LWIR has proven to be a technologically challenging endeavor.

The most intense sources currently available in the LWIR, based on CO_2_ laser technology, are limited to picosecond (>30 field cycles) durations^18^ and have progressed slowly since their early use in strong-field ionization experiments^19^. A standard method for generating femtosecond pulses in this region employs difference frequency generation (DFG), where two high frequency fields are mixed in a suitable nonlinear medium to produce a new field which can possess a much longer wavelength^20–24^. Recently, a new source based on optical parametric chirped-pulse amplification has demonstrated 200 *μ*J, ~8-cycle pulses with a MWIR wavelength of 7 *μ*m^25^, while another based on DFG has achieved sub-cycle fields at 30 *μ*J, spanning 2–9 *μ*m, but with a central wavelength of 4.2 *μ*m^26^. To date, no strong-field ionization studies have been reported with these sources due to the difficulty of achieving the required peak intensities.

In this work we demonstrate an intense, tunable light source in the LWIR possessing only 2.8 cycles under the FWHM at a central wavelength of 8.9 *μ*m. Using this apparatus we demonstrate, to the best of our knowledge, the first strong-field ionization in the LWIR with femtosecond pulses. These results utilize a collinear DFG scheme, which has several advantages. First and foremost, the technique requires only one nonlinear crystal after an optical parametric amplifier (OPA) while simultaneously delivering fields without spatial chirp (ensuring feasibility for achieving high peak intensities). Secondly, the short path length and minimal number of transmissive optics produces near transform-limited, few-cycle pulses. Finally, with a front-end based on Ti:Sapphire and an OPA, technology present in most strong-field science laboratories, this technique can be readily implemented at other facilities.

## Results

### Generation of ultrafast, long-wavelength infrared pulses

**Figure 1:**
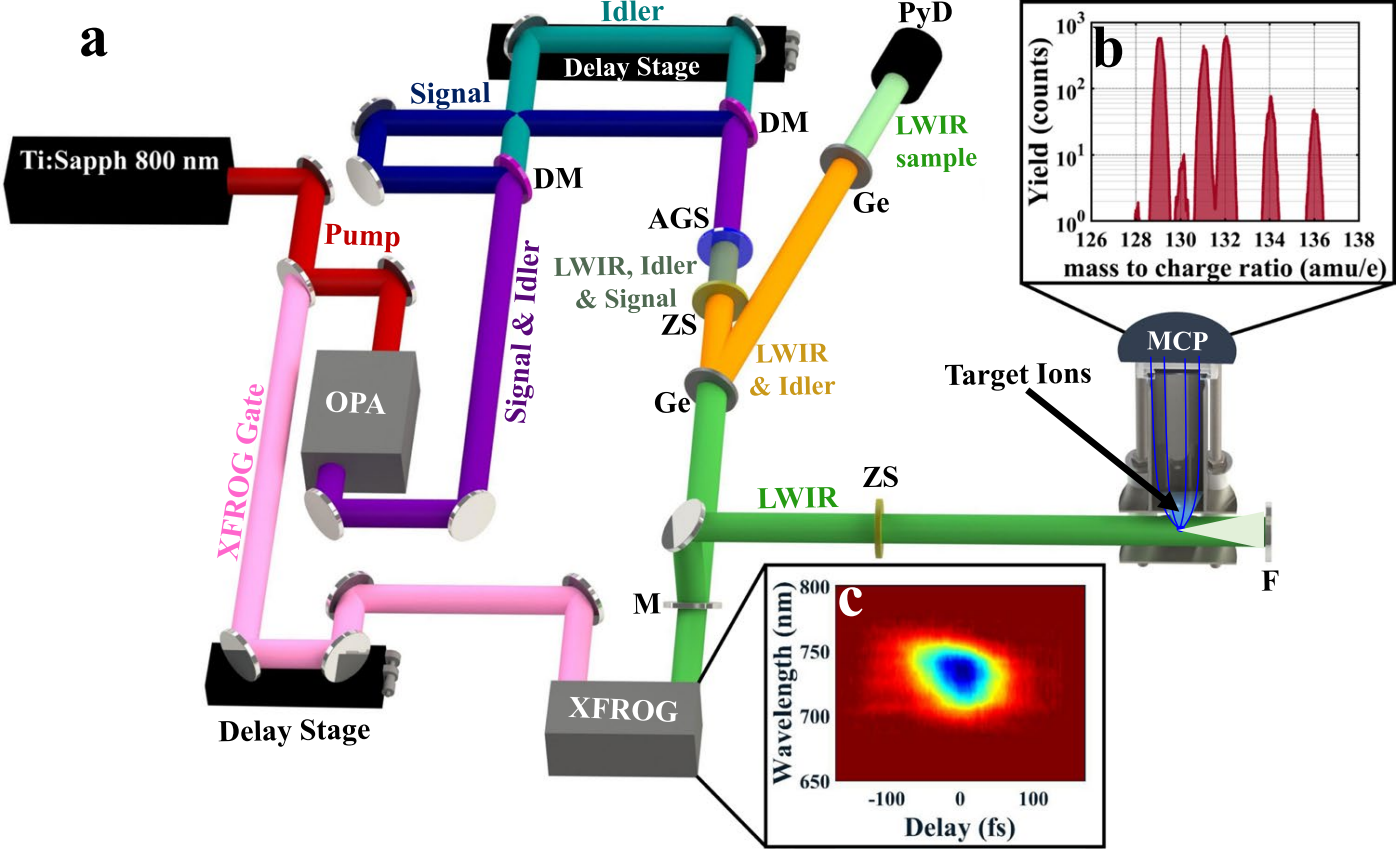
Layout for the LWIR generation, (**a**) The output of a 20 mJ Ti:Sapph 800 nm laser is separated with a beam splitter (BS) to pump an optical parametric amplifier (OPA) with 18 mJ, 26 fs pulses at 1 kHz. The OPA generates 6 mJ of signal + idler, up to 4.2 mJ of this beam are used in a dichroic mirror (DM) based interferometer to generate LWIR pulses via difference frequency generation in a AgGaS_2_(I) crystal (AGS). The OPA is filtered using a pair anti-reflective coated Zinc Selenide (ZS) and Germanium (Ge) windows. The Fresnel reflection off the Ge window is filtered with an additional Ge window so residual leakage from the LWIR beam can be recorded on a pyroelectric detector (PyD) for single-shot power tagging. A periscope sends the remaining pulse energy (>95%) into an experimental chamber with a ZS window and a −25 mm spherical concave mirror (F) to back focus the few-cycle LWIR pulse in Xenon (Xe) gas. A pair of electrostatic lenses guide the Xe ions onto a micro-channel plate (MCP) detector for time-of-flight measurements. A small portion of the remaining 2 mJ beam (pink), split prior to the OPA, is used as a gating field in the XFROG. Mirror M is removed from the periscope to couple the LWIR pulse into the XFROG for electric field characterization. Ion Time of Flight (iTOF), (**b**) A typical time of flight spectrum for Xe^+^ when ionized with 8.9 *μ*m pulse. XFROG, (**c**) A measured XFROG spectrogram for an 8.9 *μ*m laser field.

As shown in Fig. 1(a), the system uses an 800 nm, Ti:Sapphire laser to pump an OPA generating up to a combined 6 mJ of signal (1300–1450 nm) and idler (2000 nm–1730 nm) in the NIR. Additional information about the laser and OPA system can be found in Langdon *et al*.^27^. DFG between the signal and idler of the OPA is performed in an interferometer-based setup. Collinear overlap is ensured by observing the spatial chirp of the LWIR mode in the far field. This allows the beam to be focused to the smallest possible area and, thus, produce the highest peak intensities.

For DFG, we use a 1 × 1 × 0.1 cm, anti-reflective (AR) coated AgGaS_2_(I) crystal (AGS, Altechna). With this crystal, up to 4.2 mJ (signal + idler) of the OPA can be used in the DFG process. Incident energies greater than 4.2 mJ accelerate degradation of the crystal and show signs of nonlinear back-conversion. By tuning the central wavelength of the signal from 1300 nm to 1450 nm, with the corresponding idler changing from 2000 nm to 1730 nm, the central wavelength of the DFG output is tuned from approximately 3.5 *μ*m to 9 *μ*m.

**Figure 2:**
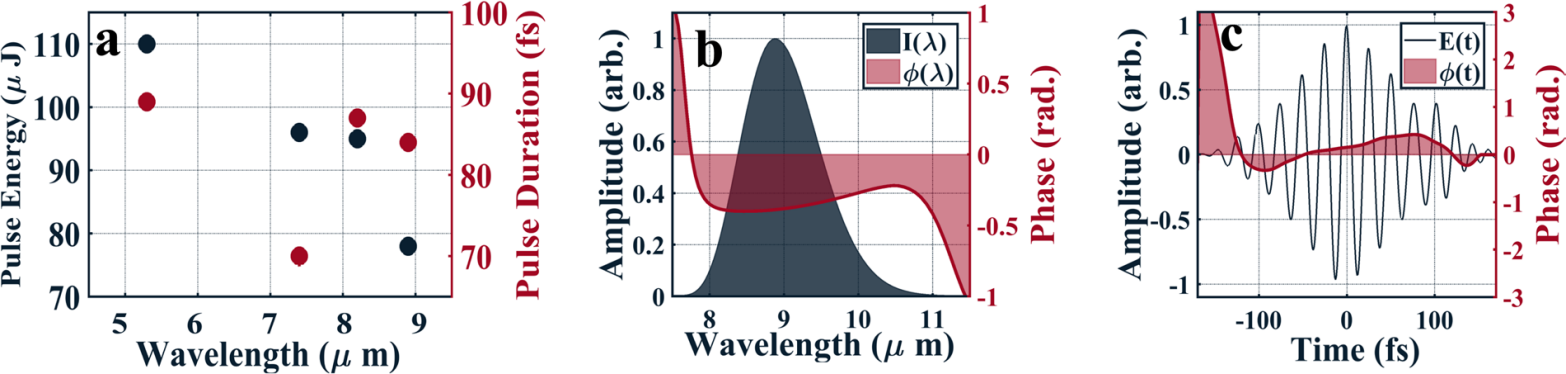
(**a**) Pulse energy (*μ*J, black) and pulse duration (fs, red) across the tuning range of the LWIR source. (**b**) Retrieved LWIR spectrum (blue) and phase (red) from XFROG measurement. (**c**) Retrieved time-domain, few-cycle electric field (black) and phase (red) measured with XFROG.

As we require a collinear arrangement for high peak intensities, the LWIR field must be separated from the higher frequency beams using a 1 mm thick, AR coated, low-pass, Germanium (Ge) filter. However, the OPA’s large fluence on the Ge surface produces a high density of free carriers that can attenuate the LWIR pulse by as much as 55%. We reduce the free carrier density by incorporating a coated, 1 mm thick, zinc selenide window before the Ge filter, which has an AR coating for the 3–12 *μ*m region that is highly reflective (≈90%) of the OPA signal. This increases the usable peak power in the LWIR by as much as a factor of 2.2 at wavelengths greater than 8 *μ*m without hindering other characteristics of the LWIR field, such as pulse duration (see section 2 of Supplementary Information). As shown in Fig. 2a, with this filtering configuration, the setup generates a maximum of 120 *μ*J at 5.3 *μ*m and up to 80 *μ*J at 8.9 *μ*m center wavelengths.

### Temporal characterization

**Figure 3:**
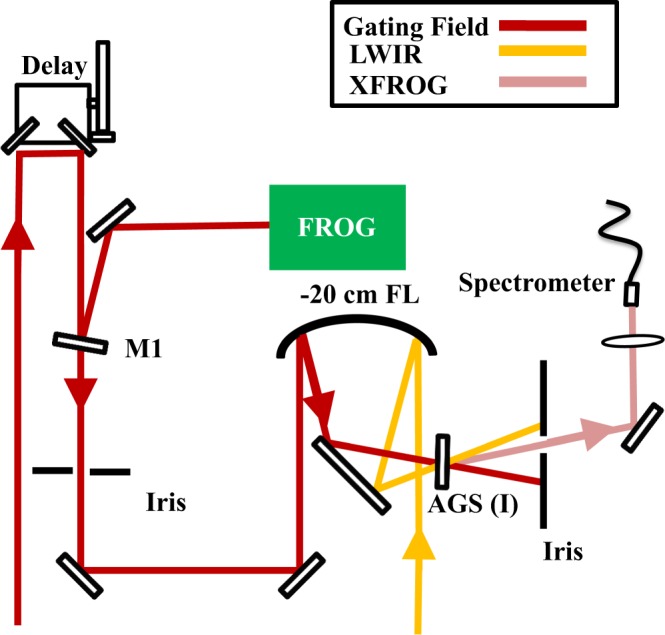
Layout for the cross-correlation frequency resolved optical gating (XFROG) pulse measurement setup. The LWIR pulse and the 800 nm gating field are sent to a 50 mm diameter, −200 mm focal length silver coated mirror to non-colinearly overlap the beam on a 8 × 8 × 0.4 mm AgGaS_2_ Type (I) crystal for sum frequency mixing. The XFROG signal is then spatially filtered with an iris and collected with a fiber and measured with an NIR-Vis spectrometer. The spectrogram is taken by recording spectra versus delay using an automated stage on the gating field. A measurement of the gating field can be performed with an additional FROG when removable mirror M1 is inserted into the beam line.

A characterization of the electric field is performed using a cross-correlation frequency resolved optical gating (XFROG) device^28^. As shown in Fig. 1(a), a portion of the Ti:Sapph pump laser is used as the gating field in the XFROG through frequency sum of the 800 nm and the LWIR pulses. The laser provides a transform-limited pulse for the OPA, so the residual beam is only slightly chirped at the XFROG generation position, which ensures a simple gating field for the retrieval algorithm. The XFROG setup is shown in Fig. 3. A spectrogram, such as the one shown in Fig. 1(c), is acquired by delaying the gating field with respect to the LWIR pulse. We find near transform-limited pulses between 70 fs to 90 fs with center wavelengths ranging between 5.3 to 8.9 *μ*m. Figure 2(a) shows the measured pulse energy and duration across this tuning range. For a field with an 8.9 *μ*m central wavelength, Fig. 2(b) shows the retrieved spectral amplitude and phase, while Fig. 2(c) shows the retrieved time-domain electric field and phase. Wavelengths greater than 6 *μ*m yield pulses with fewer than three optical field cycles under the FWHM.

**Figure 4:**
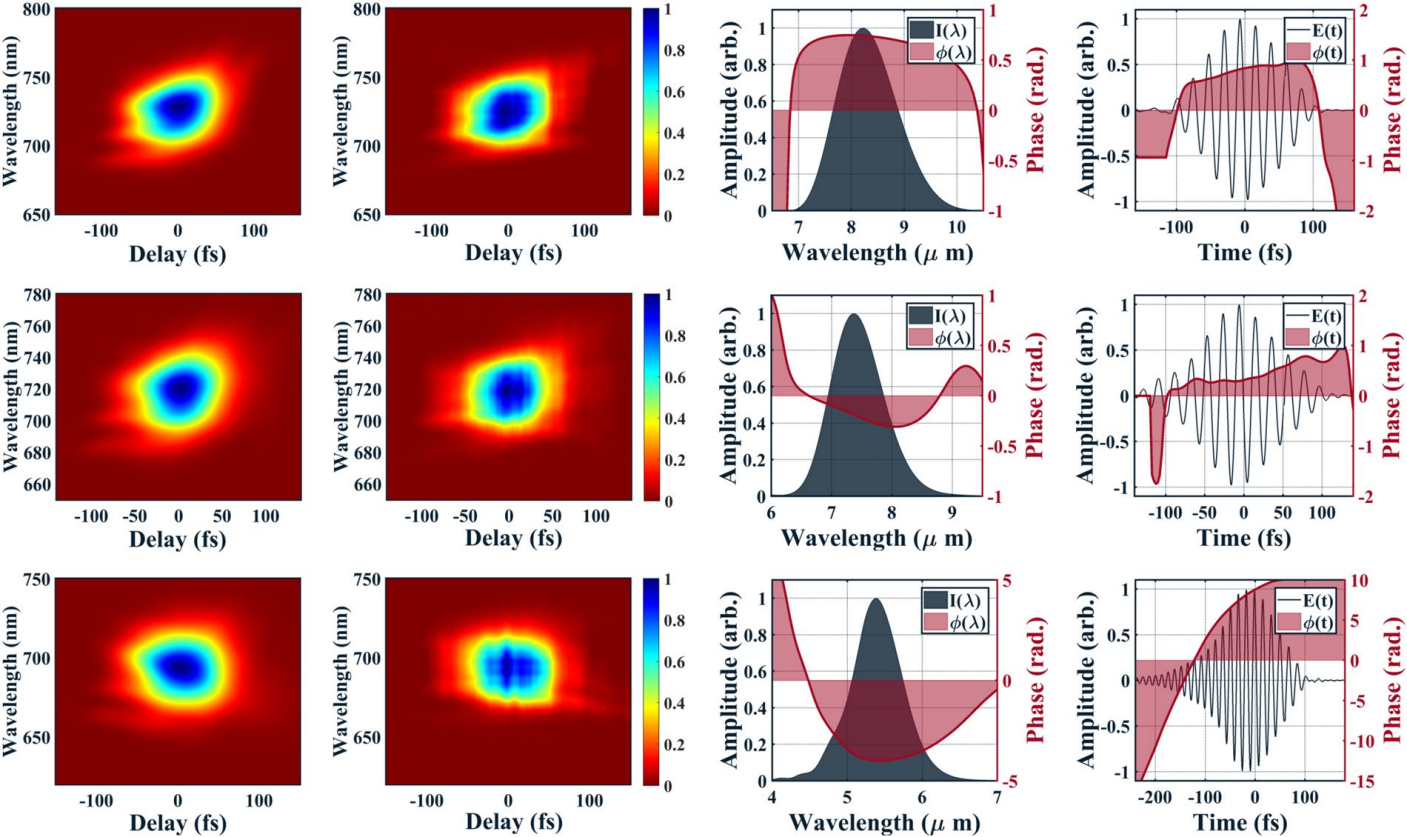
XFROG results across the range of the source. Each row represents one individual wavelength. Starting from the top row: central wavelength (1) 8 *μ*m, (2) 7.2 *μ*m, (3) 5.3 *μ*m. Each column shows, starting from the left: (1) retrieved XFROG spectrogram, (2) measured XFROG spectrogram, (3) retrieved spectra in intensity (black) with phase, (red) (4) time domain electric field (black) and phase (red).

In addition to the retrieved fields shown in 2(b) and 2(c), XFROG results over a broad range of wavelengths from our LWIR apparatus are shown in Fig. 4. The first column shows the retrieved XFROG spectrograms while the measured XFROG spectrograms are shown in the second column. The central wavelength of each spectrogram is given by the frequency sum of the LWIR and 800 nm central frequencies. The third column shows the retrieved spectra in intensity (black) with their phase (red) and the last column shows the time-domain electric field (black) and phase (red). Each row is for a different wavelength, starting with 8 *μ*m on the top row and down to 5.3 *μ*m on the final row.

We plot the electric field for a carrier envelope phase (CEP) of zero for Fig. 2c and the third column of Fig. 4. This choice was made to emphasize the few-cycle nature of the fields yielded at each of the central wavelengths. The XFROG characterization is insensitive to the value of the CEP and the source was not run in a mode to provide CEP stability to the LWIR fields. In the future, we can expect to deliver CEP stable LWIR fields by actively stabilizing the CEP of the Ti:Sapph system^27^. This, combined with the OPA’s passively CEP stable idler^29^, provides a straightforward route to phase stable LWIR fields.

### Strong-field ionization in the long-wave infrared regime

**Figure 5:**
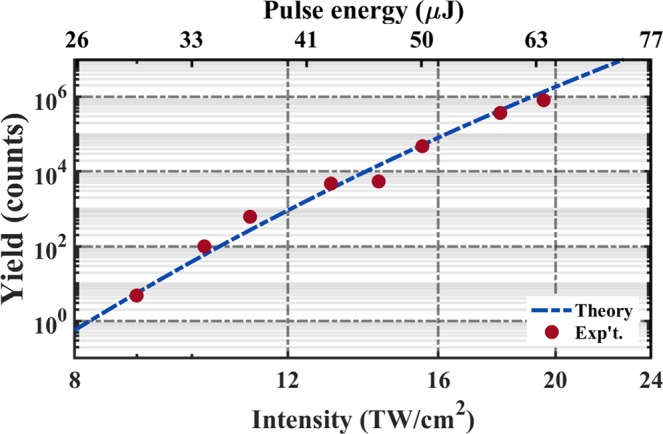
Ionization of noble gases. Measured yield of Xe^+^ for *λ* = 8 *μ*m versus the measured pulse energy (top axis) and fitted intensity (bottom axis). Error bars for the yield were calculated and found to be smaller than the marker size. The experimental yield is fitted to a full Perelomov-Popov-Terent’ev^31^ simulation, as described in the text.

As proof of this source’s ability to deliver intense fields across a large wavelength range, we measure the strong-field ionization of xenon (Xe) atoms at 8.9 *μ*m, 8 *μ*m, and 5.3 *μ*m (not shown) using an ion time-of-flight (iTOF) apparatus. Figure 1(a) shows the experimental setup. In addition to the ion yields, we monitor single-shot pulse energy fluctuations using a pyroelectric detector (PyD). Pulse energy control is achieved by employing a fused-silica based, variable neutral density filter in the OPA signal arm of the LWIR interferometer. As this could cause the spatio-temporal qualities to change, the role of *χ*^(3)^ processes in the AGS crystal was investigated and, to our best knowledge, these processes are not present in this system. The iTOF was designed to support the 2.5 cm back-focus required for the experiment, otherwise it is a common arrangement with an extractor, repeller, and drift tube. Figure 1(b) shows an iTOF measurement for Xe^+^ ionized using a *λ* = 8.9 *μ*m pulse which clearly shows all the abundant Xe isotopes. No higher charge states of Xe were observed. Further details of the detection system are found in the Methods section. Figure 5 shows the measured ion yield of Xe^+^ as a function of input pulse energy (top axis) in log-log scale for *λ* = 8 *μ*m. These results represent, to the best of our knowledge, the first strong-field ionization results, with femtosecond pulses, in the LWIR. The wavelength dependence of ionization versus intensity will be presented in an upcoming publication.

To better understand the ionization versus intensity results at 8 *μ*m, we undertook two measures for determining the intensity range. First, we determined the beam waist at the gas target in the iTOF using a scanning slit and a thermopile detector. The measurement, described in section 3 of the supplement, determined that the setup could achieve a 1/e^2^ radius waist of 30 *μ*m for the back-focusing system in Fig. 1. Taken together with our pulse energy and XFROG measurements, this corresponds to a maximum peak intensity of 50 TW/cm^2^. Further improvement in the beam quality, such as using a spatially filtered OPA^30^, can dramatically improve the focusing characteristics without sacrificing the peak power. Our second route to determining the peak intensity implemented a fit of our data to the Perelomov-Popov-Terent’ev (PPT)^31^ formulation of strong-field ionization theory. PPT was recently shown to accurately describe experimental results for multi-cycle pulses from 400 nm to 3.9 *μ*m^32^. Fitting our data, shown as the dashed blue line in Fig. 5, suggests a peak intensity of 20 TW/cm^2^ (bottom axis), which is a factor of 2.5 lower than the estimate from our beam waist measurement. As the source delivers few-cycle fields, the effect of a varying CEP was also considered and found to have an insignificant change in the overall ionization rate. If we consider the intensities predicted from our fit to PPT, we observe Xe^+^ ionization at a Keldysh parameter^33^ value of 0.23 and in a region where the HHG cutoff^19^ is nearly 400 eV. With more attention to the focusing quality of the source, it should be possible to reach the 100 TW/cm^2^ mark.

## Conclusion

We have demonstrated the generation of intense, few-cycle pulses in the LWIR tunable from 5.3 *μ*m to 8.9 *μ*m. Our collinear DFG scheme maintains broadband transform-limited pulses in this wavelength range. We experimentally measured the pulse durations to be between 70 to 90 fs, depending on the wavelength, delivering a 5.8 cycle field at 5.3 *μ*m and 2.8 cycles at 8.9 *μ*m. Over this entire wavelength range, the source can be focused on target to peak intensities high enough to induce strong-field ionization in Xe. To the best of our knowledge, these are the longest wavelengths that a femtosecond pulse has been used to ionize neutral atomic targets, paving the way for many new and exciting studies in the intense long-wave infrared regime.

## Methods

### Electric Field Characterization using Cross Correlation Frequency Resolved Optical Gating

Our XFROG setup employs sum frequency mixing between the LWIR and a residual 800 nm beam from the Ti:Sapphire laser. The sum frequency signal between the LWIR and the gate is generated in a non-collinear arrangement. This ensures that the 700–740 nm XFROG signal can be spatially separated from the pump beam. The 800 nm gating field and the LWIR beam are incident on a 50 mm diameter, −200 mm focal length silver coated spherical mirror. The beams are placed on opposite sides of the mirror so that they non-collinearly overlap in a 8 × 8 × 0.4 mm AgGaS_2_ Type (I) crystal (AGS). This allows the weak sum frequency beam to be spatially separated from the much stronger gating field using an iris. After collecting the generated sum frequency signal in a spectrometer, a spectrogram (like the ones shown in column 2 of Fig. 4) can be collected using a delay stage. In order to retrieve the unknown field from this spectrogram, we must use a well-characterized gating field. We perform this by inserting mirror M1 (see Fig. 3) and sending the gating field to be characterized with a second harmonic generation frequency resolved optical gating (SHG-FROG) instrument. The gating field is first characterized using the FROG 3.2.2. software from Femtosoft Technologies and generates a retrieved electric field. With this, an XFROG retrieval can then be computed using the same software to determine the electric field characteristics of the LWIR field. To ensure that the crystal can support enough bandwidth, the XFROG results were tested with thicker a 8 × 8 × 1 mm AgGaS_2_ crystal and no difference was observed as compared to the 0.4 mm thick crystal. Further details on the XFROG measurements are given in part 1 of the Supplementary Information.

### Filtering

When a 1 mm thick ZS window is inserted before the 1 mm thick Ge window in the path leading out of the AGS crystal, we find that the available energy for the LWIR pulse increases approximately 20% (220%) for a 5 *μ*m (8 *μ*m) pulse, as compared to using only the Ge window. A series of additional tests were performed to determine the nature of this observation. The analysis determined that the transmission of LWIR light through Ge increases as the fluence of the OPA signal decreases on the Ge surface. This can be explained by the OPA signal (0.85–0.9 eV) performing a one photon transition to Ge’s direct band gap (0.8 eV), which subsequently allows LWIR photons to be lost to free carrier absorption (FCA). This is further supported by two facts. First FCA rates increase wavelength squared, making 8 *μ*m more susceptible than 5 *μ*m, which is what we see. Secondly, the AR coating on the ZS window reflects more than 90% of the OPA signal, which removes the first step of the proposed mechanism.

### Time-of-Flight measurements

All ionization experiments were performed using an ion time-of-flight (iTOF) mass spectrometer. The chamber background pressure was 1⋅10^−7^ torr, consisting mainly of common atmospheric gases. The Xe gas sample was introduced by backfilling the vacuum chamber to a pressure of approximately 2 × 10^−5^ torr for the studies at 8.0 *μ*m. For each intensity point, a time-of-flight spectrum is recorded for 1.2 million laser shots, which requires 20 minutes. Ion yields shown in Fig. 5 are normalized to the Xe pressure in the chamber when the measurement was performed. A constant extraction potential was maintained for all runs with a 2 kV voltage applied to the repeller and −2.2 kV applied to the electrostatic lens system. Ions generated in the interaction region of the laser are guided to a micro-channel plate (MCP) detector. The front of the MCP was held at −2.0 kV to accelerate the ions to an adequate energy for MCP activation. The MCP signal was amplified using a fast amplifier and processed by a constant fraction discriminator (CFD). A time to digital converted was then used to record the ion time of flight for each individual ion with respect to a photodiode signal.

Simultaneously, a pyroelectric detector (PyD) was used to the measure a small fraction of the LWIR beam to allow for single-shot pulse energy tagging. For this tagging, we used the Fresnel reflection from a Ge window (See Fig. 1(a)) filtered with an additional set of Ge windows to record a small (<*μ*J) amount of LWIR leakage on the PyD. This allows for single-shot monitoring of the LWIR pulse energy by recording the peak of the electronic signal with an analogue to digital converter synchronously with the iTOF signal. The single-shot PyD was calibrated for each wavelength, by measuring the average power with a thermopile sensor and also taking into account the in-coupling window of the vacuum chamber. Finally, a periscope steers the main LWIR into the iTOF apparatus and is back focused inside the chamber with a spherical concave mirror of 25 mm focal length.

## Supplementary information

Supplementary information
