# Supplementary material for: An intense, few-cycle source in the long-wave infrared
Source: Sci Rep. 2019 Apr 12;9:6002. doi: 10.1038/s41598-019-42433-1 (PMC6461661; doi:10.1038/s41598-019-42433-1)
Supplement: Supplementary file 1 — Supplementary information [file 41598_2019_42433_MOESM1_ESM.pdf]

An intense, few-cycle source in the long-wave infrared

Derrek J. Wilson[1,2], Adam M. Summers[1], Stefan Zigo[1], Brandin Davis[1,3], Seyyed-Javad Robatjazi[1], Jeff Powell[1], Daniel Rolles[1], Artem Rudenko[1], Carlos A. Trallero-Herrero[1,3]

[1] J. R. Macdonald Laboratory, Kansas State University, 116 Cardwell Hall, Manhattan, KS 66506

[2] Advanced Laser Light Source and few-cycle Inc., Institut Nationale de la Recherche Scientifique, 1650 Boul. Lionel-Boulet, Varennes, QC J3X 1P7

[3] Department of Physics, University of Connecticut, 2152 Hillside Road, Unit 3046, Storrs, CT 06269

1 Electric Field Characterization using Cross Correlation Frequency Resolved Optical Gating

Cross-Correlation Frequency Resolved Optical Gating (XFROG) is a cross-correlation optical measurement technique which is favorable when an autocorrelation signal is difficult to measure or produce [1]. In the case of our ultrafast LWIR pulses, a frequency resolved measurement of the second harmonic (SH) field requires a HgCdTe or pyroelectric detector based spectrometer, each of which pose their own technical limitations. On the other hand, an XFROG, employing the sum frequency (SF) of 800 nm pulsed directly from our Ti:Sapph laser + LWIR pulses, results in a signal with a center wavelength between 650 and 725 nm. This signal is both measured with a readily available, silicon detector based spectrometer and is visible by eye and thus easily optimized in the laboratory setting.

In XFROG a known gating pulse is used to measure the unknown pulse. Therefore the gating field must be independently measured or characterized. We accomplish this using a standard second harmonic generation frequency resolved optical gating (SHG-FROG) device. Figure 1 shows an example of the retrieved gating field using SHG-FROG. For an accurate XFROG retrieval, this measurement is setup so that the path length to the BBO in the SHG-FROG is within 1 cm of the path length to the XFROG's AGS crystal. The paths for both SHG-FROG and XFROG also use the same transmissive optics (e.g. neutral density filter, iris with same inner diameter and position). This ensures that the field measured in the SHG-FROG has identical characteristics to the gate for XFROG. From the figure we can observe that while there is a second-order phase present, the pulses are close to transform-limited with amplitude and phases in both the temporal and spectral domain that are simple.

As an accuracy confirmation of our XFROG method, we demonstrate the precision by comparing the field characterization of the OPA's signal beam using SHG-FROG and XFROG. At a central wavelength of 1340 nm we can use exactly the same optical components and detectors for both setups. Referring to figure 1 in the main letter, we see that by blocking the OPA idler and removing AGS, zinc selenide (ZS), and germanium (Ge) in the initial path of LWIR generation, we can perform XFROG on the OPA signal while maintaining an almost identical optical path as the LWIR. Characterization results of 1340 nm pulses using SHG-FROG versus XFROG are shown in figure 2. We used the same BBO crystal in both cases. Figures 2(a) and 2(b) are the measured and retrieved spectrograms for the OPA signal using SHG-FROG, respectively. Figures 2(c) and 2(d) are the measured and retrieved spectrograms using XFROG.

The comparison between the two methods in time domain is shown in figure 2(e). The dashed lines

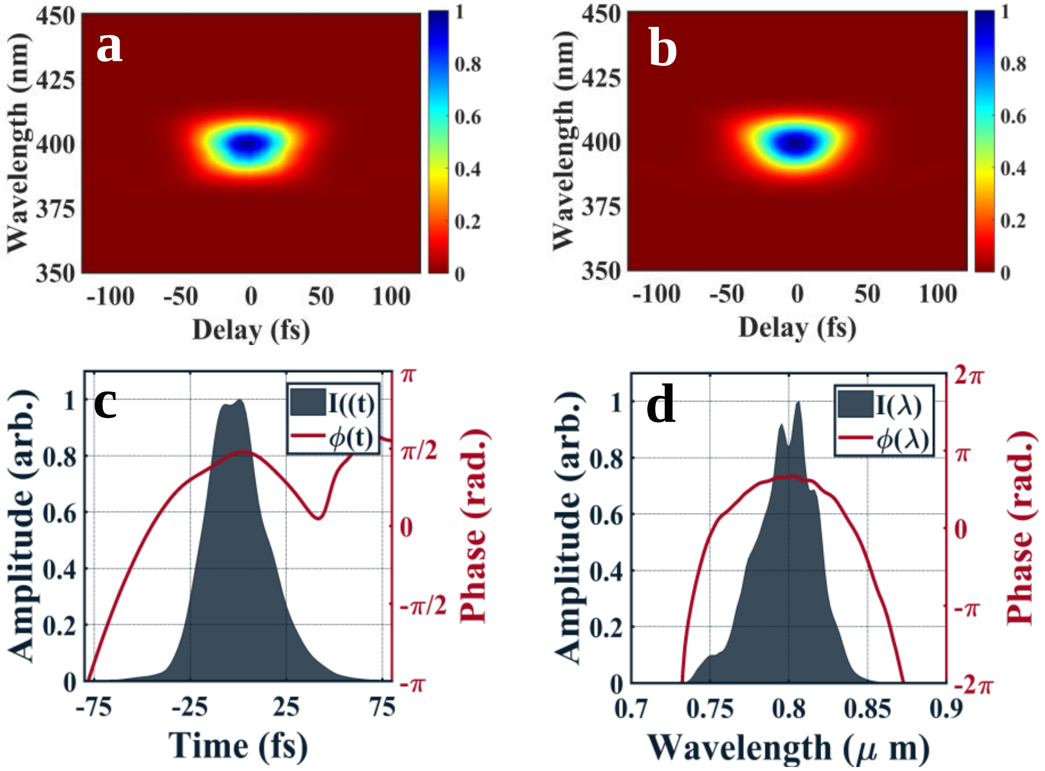

Figure 1: Retrieved electric field for the 800 nm pulses used as gating in the XFROG. **(a)** Measured FROG spectrogram. **(b)** Retrieved FROG spectrogram. **(c)** Time-domain intensity (shaded), and field phase (red). **(d)** Retrieved spectral intensity (shaded) and field spectral phase (red).

represent amplitude and the dotted lines represent phase. In black are the results for SHG-FROG while the XFROG measurements are shown in red. Figure 2(f) shows the retrieved spectral intensity. As before, the dashed lines are amplitude while dotted are phase, and XFROG/SHG-FROG results are shown in red/black respectively. From the figures, we see that the overall shape of the intensity envelope is almost identical yielding durations of about 40 femtoseconds for XFROG versus 42 femtoseconds for FROG. The time-domain phases are also very close under the envelope and diverge only at delays where the field is almost non-existent. Both methods show a dominant second-order chirp present in their retrievals. Spectral reconstruction shows the same trend as the time-domain except for the fact that the XFROG retrieval yields a slightly narrower bandwidth compared to SHG-FROG.

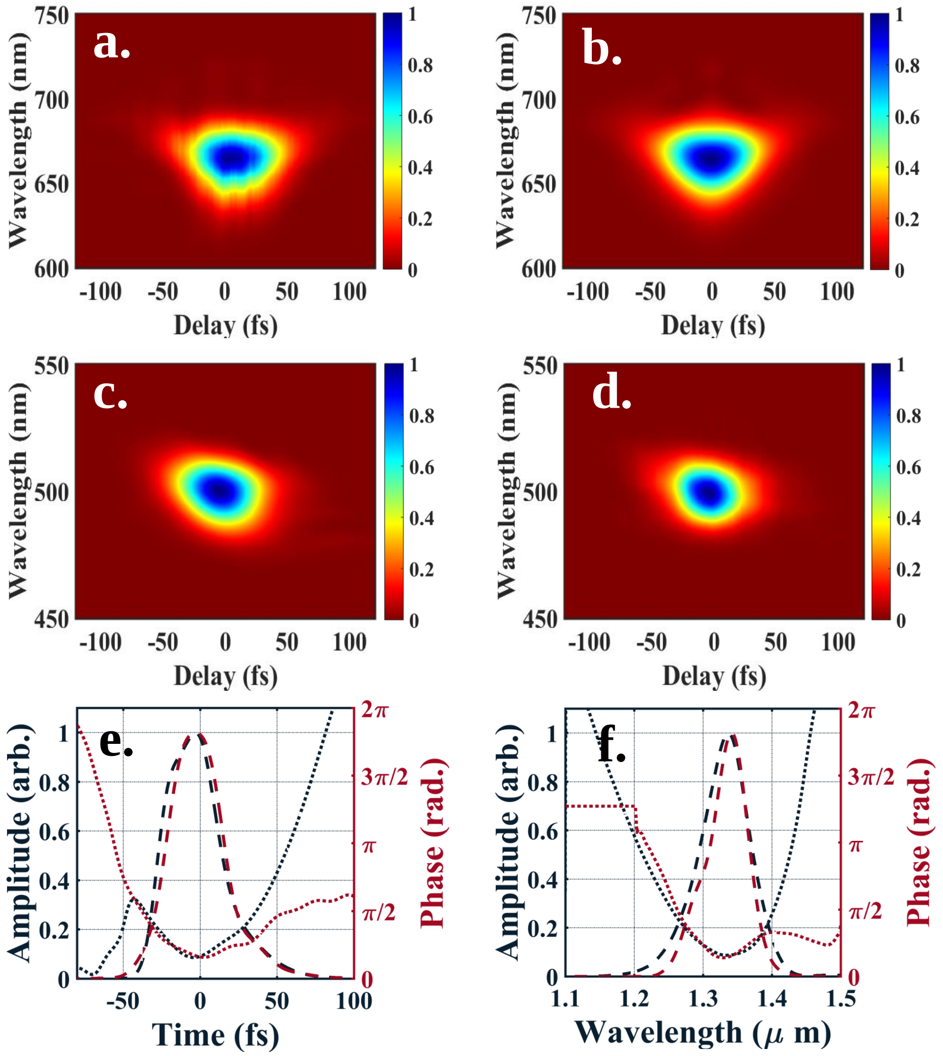

Figure 2: Comparing XFROG and FROG on the OPA signal (1340 nm). **(a)** Measured FROG spectrogram. **(b)** Retrieved FROG Spectrogram. **(c)** Measured XFROG spectrogram. **(d)** Retrieved XFROG spectrogram. **(e)** Retrieved time domain intensity envelope (dashed lines) and phase (dotted lines). Lines colored black are for the FROG and red lines are for the XFROG. **(f)** Retrieved spectra in intensity (dashed lines) with phase (dotted lines). Lines colored black are for the FROG and red lines are for the XFROG.

2 The role of free carriers in optical filtering of intense long wave infrared pulses

To achieve the highest peak intensity with a DFG-based source it is necessary to use a collinear geometry for the two high frequency fields[2;3], as non-collinear arrangements yield a spatial chirp which increases both the pulse duration and the beam waist at the focus of the generated field. A collinear geometry, however, does not allow for spatial filtering of the generated field[4] and thus must use material-based filtering techniques. When removing NIR-VIS from the MWIR-LWIR, the primary choice of filter is germanium (Ge), as it possesses a suitable absorption spectrum which, when combined with appropriate anti-reflective coatings, permits efficient separation of these two frequency regimes.

However, when the fluence of the fields on the Ge surface are high enough, we find that additional effects arise causing the overall transmittance of the LWIR to be lowered. As was discussed in the main letter, it was observed that inserting a zinc selenide (ZS) filter before the Ge filter increased the measured pulse energy, while inserting ZS after Ge yields a 3-5 % drop in the measured value. Since we are in the high-fluence regime, it is important to consider the presence of nonlinear effects in Ge. We examine this by performing two studies targeting transmission as a function of peak intensity, a main driver in nonlinear processes.

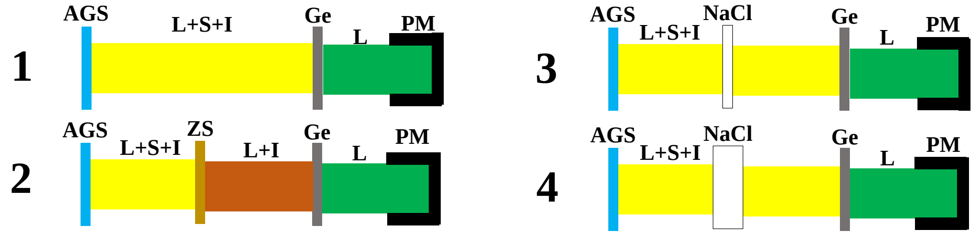

Figure 3: Four combinations were used to determine the effects of peak intensity, controlled through chirp in the collinear filtering geometry. Zinc selenide and NaCl can produce the same amount of negative dispersion in the LWIR. AGS - $AgGaS_2$(I) crystal, ZS- zinc selenide window, Ge- germanium window, NaCl - sodium chloride window, PM - power meter, L- LWIR beam, S- OPA signal, I- OPA idler.

The first study controlled the pulse duration (and thus intensity) by chirping the field with the dispersion of bulk materials. Zinc selenide has a negative group velocity dispersion of - 17 fs^2/mm at 5 μm and -519 fs^2/mm at 8 μm. By using another material with negative GVD, such as sodium chloride (NaCl) (- 130 fs^2/mm at 5 μm, -630 fs^2/mm at 8 μm), we can vary the pulse duration of the LWIR field inside Ge. If the nonlinear process is driven by the peak intensity of the LWIR, then this chirping effect should be material independent and, therefore, present with both ZS and NaCl. Figure 3 shows the layout for determining the role of chirp in the transmission of LWIR pulses. We start by generating a LWIR field in our AGS crystal and used four combinations of filtering the OPA's signal and idler.

Configuration	5 μm energy (μJ)	8 μm energy (μJ)
1	90	37
2	106	80
3	75	28
4	63	30

Table 1: Measured pulse energy for the four filtering combinations shown in Fig. 3.

Transmission measurements were done with a thermopile sensor. The first combination is a single, 1 mm thick, Ge window with a 3-12 μm anti-reflective (AR) coating and acts as our base reference. The second combination includes a 1 mm thick, ZS window, also with a 3-12 μm AR coating, before Ge. The third and fourth combinations replace ZS with a 2 mm and 5 mm thick, uncoated NaCl window, respectively.

Table 1 shows the transmission results for the four configurations shown in figure 3 for 5 μm and 8 μm. It is clear from the table that using a ZS window in front of Ge is the best possible combination, despite having a much shorter (and more intense) LWIR field than either combination with NaCl. While the enhancement at 5 μm is roughly 20 %, the enhancement at 8 μm is much larger at 220 %. This large enhancement can not be explained by the presence of Fresnel losses in the NaCl windows, which are only ~4-5% (for each surface) in this wavelength range

The main difference between ZS and NaCl is that the low-pass, AR coating on ZS blocks over 90 % of the OPA signal, while the NaCl windows pass most of the OPA light. Thus, we suspected that the OPA signal causes the absorption of LWIR inside Ge. We investigated the role of the OPA signal's fluence on Ge with the setup in figure 4. A -50 mm uncoated CaF_2 lens expands the three beams exiting the AGS

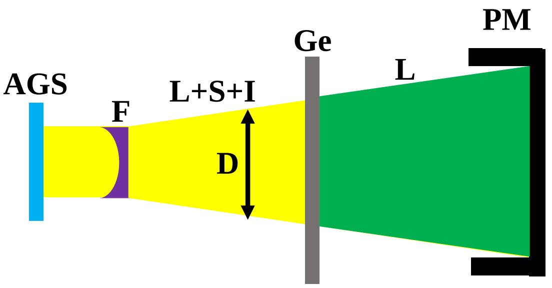

Figure 4: Setup for measuring the effect of fluence on the germanium (Ge) window. AGS - $AgGaS_2$ crystal, F- uncoated -50 mm CaF_2 lens, Ge- AR coated 50 mm diameter germanium window, PM- large area thermopile based sensor, L- LWIR, S- OPA signal, I- OPA idler.

Beam diamater (cm)	5 μm energy (μJ)	8 μm energy (μJ)
1	60	30
1.5	70	40
2	90	50
2.5	110	60
3.5	120	70

Table 2: Measured pulse energy for two wavelengths as a function of beam diameter on Ge window surface as shown in the setup in figure 4.

crystal. We used a large area thermopile sensor to measured the transmitted energy. Fluence at the Ge window is controlled by scanning the window's position in the direction of laser propagation.

Table 2 shows the results for this fluence scan, as a function of spot size at the Ge window, for the same wavelengths as table 1. Due to the large error in the thermopile sensor, measured pulse energies are to the nearest 10-20 μJ level and should only be considered an estimate. Nonetheless, the results clearly indicate that a larger area beam, and thus a smaller fluence, on the Ge window surface increases LWIR transmission.

Combined, the results of tables 1 and 2 indicate that LWIR can be attenuated with a high fluence of the OPA on the Ge window. Ge has a direct band gap of 0.8 eV and an indirect band gap at the L point of 0.66 eV, allowing it to strongly absorb all light from the OPA but particularly the signal with wavelengths smaller than 1500 nm. Thus, we suspect that the transmission of LWIR is lowered via a two-photo-absorption process consisting of two resonant (or near resonant) transitions. The larger OPA signal photons couple states from the valence to the conduction band while the LWIR photons couple states within the conduction band. By blocking the OPA signal, we reduce the free carrier density, reducing the rate of the LWIR absorption, and increasing the overall transmittance.

An electron in a semiconductor's conduction band more readily absorbs lower energy photons because of the larger density of states. Wavelength dependence can be understood by a simple derivation for free carrier absorption rate using the Drude Model[5;6],

$$\alpha = \frac{\lambda^2 e^3 N_c}{4\pi^2 c^3 \epsilon_0 n_r m_e^2 \mu_c}, \tag{1}$$

where λ is the wavelength of light being absorbed, e is the electron charge, N_c is the carrier density, c is the speed of light, ϵ_0 is the permittivity of free space, n_r is the refractive index, m_e is the electron mass, and μ_c is the carrier mobility. Notice that, in the equation, the carrier rate increases with λ^2 which is consistent with our findings that longer wavelengths are more readily attenuated, as shown in table 1.

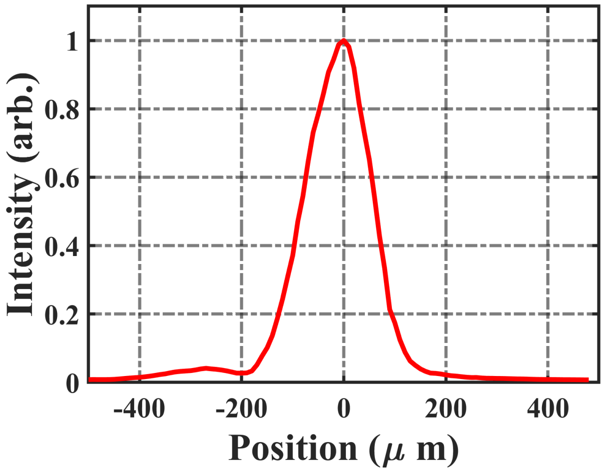

Figure 5: Measurment of the beam waist with a 40 μm wide scanning slit using a 5 cm off-axis parabolic focusing mirror. Assuming a Gaussian waist, a convolution was extracted from the data retrieves a 60 μm 1/e^2 radius.

3 Beam waist measurement

As the highest achievable peak intensity goes linear with pulse energy and duration, but inverse square with beam waist, it is useful to know the focusing quality of a light source. In an effort to have a comparison with our fitting to ionization theory, we performed a beam waist measurement. This is difficult in the LWIR, as detectors in this region have a number of limitations, including poor signal-to-noise ratio, sensitivity, and/or cost. We measured the beam waist of the LWIR pulse using an off-axis parabolic mirror with a 5 cm focal length, a 40 μm wide scanning slit, and a thermopile sensor. The slit is placed at the focus of the beam on a translation stage and is scanned with 10 micron transverse steps. At each step, the thermopile is allowed to reach thermal equilibrium before moving to the next position. Care was taken to ensure the slit was not damaged near the focus by attenuating the pulse energy. The scan was allowed to go in an appreciable distance in $\pm Z$ scans to ensure that the beam waist was thoroughly sampled. To extract the beam waist from our measurement, we assume its possesses a Gaussian profile and perform a numerical convolution with the slit as a mask. The Gaussian profile minimizing the error between the computed and measured convolution retrieves a beam waist of 60 μm 1/e^2 radius. If we assume that the AGS crystal's aperture is the 1/e^2 beam width before focusing (10 mm), then the measured beam waist is a factor of 2.4 larger than the Gaussian optics prediction. Extrapolating this to the case of the 2.5 cm back-focus in the iTOF, then the maximum permissible peak intensity is predicted to be 60 TW/cm^2, which is a factor of three greater than predicted with the ionization fittings.

References

[1] S. Linden, J. Kuhl, and H. Giessen, Opt. Lett. **24**, 569 (1999).

[2] A. A. Lanin, A. A. Voronin, E. A. Stepanov, A. B. Fedotov, and A. M. Zheltikov, Opt. Lett. **39**, 6430 (2014).

[3] H. Liang, P. Krogen, Z. Wang, H. Park, T. Kroh, K. Zawilski, P. Schunemann, J. Moses, L. F. DiMauro, F. X. Kärtner, and K.-H. Hong, Nature Communications **8** (2017).

[4] A. Sell, A. Leitenstorfer, and R. Huber, Opt. Lett. **33**, 2767 (2008).

[5] C. H. Henry, R. A. Logan, and K. A. Bertness, Journal of Applied Physics **52**, 4457 (1981).

[6] C. Y. Tsai, C. Y. Tsai, C. H. Chen, T. L. Sung, T. Y. Wu, and F. P. Shih, IEEE Journal of Quantum Electronics **34**, 552 (1998).

[7] P. B. Chapple, Optical Engineering **33**, 2461 (1994).
